# The impact of data from remote measurement technology on the clinical practice of healthcare professionals in depression, epilepsy and multiple sclerosis: survey

**DOI:** 10.1186/s12911-021-01640-5

**Published:** 2021-10-13

**Authors:** J. A. Andrews, M. P. Craven, A. R. Lang, B. Guo, R. Morriss, C. Hollis, The RADAR-CNS Consortium

**Affiliations:** 1 NIHR MindTech MedTech Co-operative, Institute of Mental Health, University of Nottingham, Triumph Road, Jubilee Campus, Nottingham, NG7 2TU UK; 2 Mental Health and Clinical Neurosciences, School of Medicine, University of Nottingham, Nottingham, UK; 3 Human Factors Research Group, Faculty of Engineering, University of Nottingham, Nottingham, UK; 4 NIHR Nottingham Biomedical Research Centre, University of Nottingham, Nottingham, UK; 5 ARC-EM, School of Medicine, University of Nottingham, Nottingham, UK; 6 Kings College London, London, UK

**Keywords:** Smartphone apps, Healthcare professionals, Depression, Multiple sclerosis, Epilepsy, Remote measurement technology, Survey

## Abstract

**Background:**

A variety of smartphone apps and wearables are available both to help patients monitor their health and to support health care professionals (HCPs) in providing clinical care. As part of the RADAR-CNS consortium, we have conducted research into the application of wearables and smartphone apps in the care of people with multiple sclerosis, epilepsy, or depression.

**Methods:**

We conducted a large online survey study to explore the experiences of HCPs working with patients who have one or more of these conditions. The survey covered smartphone apps and wearables used by clinicians and their patients, and how data from these technologies impacted on the respondents' clinical practice. The survey was conducted between February 2019 and March 2020 via a web-based platform. Detailed statistical analysis was performed on the answers.

**Results:**

Of 1009 survey responses from HCPs, 1006 were included in the analysis after data cleaning. Smartphone apps are used by more than half of responding HCPs and more than three quarters report that their patients use smartphone apps or wearable devices for health-related purposes. HCPs widely believe the data that patients collect using these devices impacts their clinical practice. Subgroup analyses show that views on the impact of this data on different aspects of clinical work varies according to whether respondents use apps themselves, and, to a lesser extent, according to their clinical setting and job role.

**Conclusions:**

Use of smartphone apps is widespread among HCPs participating in this large European survey and caring for people with epilepsy, multiple sclerosis and depression. The majority of respondents indicate that they treat patients who use wearables and other devices for health-related purposes and that data from these devices has an impact on clinical practice.

**Supplementary Information:**

The online version contains supplementary material available at 10.1186/s12911-021-01640-5.

## Introduction

The easy availability and reduced cost of mobile and wearable technologies is enabling individuals to record more data about their physical and mental health than ever before. Digital health smartphone apps (software installed and run on smartphones) are known to be used for a variety of healthcare-related purposes by both patients and clinicians. For example, a multi-national online survey found that diabetes apps are used by over half of those with type 1 and one third of those with type 2 diabetes [1]. In a recent US study, just under half of survey respondents living with hypertension reported using apps to help manage it [2]. In relation to central nervous system disorders, their use has been explored in the management of depressive and bipolar disorders for mood monitoring and contextual prompting (‘THINC-IT’ [3], ‘True Colours’ [4], ‘Wear-IT’ [5]), and systematic reviews of depression apps in 2015 and 2020 identified digitized therapeutic intervention, symptom tracking and psychoeducation as main functions of apps available for this market [6, 7]. In epilepsy, apps are available for a range of purposes, including to enter details of seizures to create a record (e.g. ‘Seizure Tracker’) and to notify an emergency contact if a person living with epilepsy does not respond to a daily prompt (‘Snug Safety’ app). A systematic review of the Apple app store for epilepsy or seizure-related apps also identified education and seizure trigger recording as functions of the available apps [8]. Smartphone apps for multiple sclerosis include functions such as medication reminders and symptom trackers, in addition to assessment of function through games and questionnaires [9].

There are also a wide variety of apps available which are targeted at health care professionals (HCPs) [10], including apps providing clinical guidelines [11], apps with clinical task specific calculators [12], prescribing apps [13], and apps providing the ability for HCPs to communicate with each other and/or with patients and to inform decision-making. Both digital health apps aimed at patients and apps aimed at HCPs can be subject to regulation, depending on their intended purpose, as they may be designated ‘software as a medical device’ [14].

A large variety of off-the-shelf wearable sensing devices are commercially available, aimed at the public for the purpose of monitoring health and wellbeing. These include electronic wristbands, pedometers and devices designed for specific conditions such as epilepsy seizure detection wristbands. Many of these interface with smartphone apps to provide greater functionality. Wearable sensing devices allow their wearers to monitor many aspects of their daily lives, including steps taken, heart rate, sleep quality and duration, and exercise. As such, their use is generally for the ‘passive’ collection of data, without the user’s active input. Recently, their use as tools for monitoring patients with central nervous system disorders has garnered interest [15]. Prior work has shown that connected digital products are used increasingly in clinical trials [16], demonstrating their value as tools to accurately record aspects of patient functioning. Patients themselves have a variety of views on the use of apps and wearables for monitoring specific conditions, including both benefits and challenges [17, 18].

When wearable devices and smartphone apps are used to collect data on human behaviour and physical parameters, these information-gathering tools are collectively described as remote measurement technology (RMT). The RADAR-CNS project, funded by Horizon 2020 and the Innovative Medicines Initiative, explores the potential of RMT in the clinical management of epilepsy, MS and depression. The consortium consists of a team of researchers, clinicians and a patient advisory board of individuals with lived experience of epilepsy, multiple sclerosis (MS), or depression (www.radar-cns.org). Project outputs to date have provided insight into the potential benefits of incorporating RMT in clinical pathways, via qualitative surveys of patients and healthcare professionals within the consortium [19] and interviews with HCPs outside the consortium [20]. In these small studies, HCPs and patients envisaged multiple ways that RMT could permit both ‘active’ (direct user input of data, such as mood diaries) and ‘passive’ (collection of data without the requirement of the user to input data, e.g. step count, GPS location) collection of data to remotely assess patients with these conditions.

This paper adds to this body of work by exploring the experiences of a much larger number of HCPs working with patients with epilepsy, MS, or depression outside the consortium. In studies relating to health care technology, exploring views from a wide range of health care professionals working across different job roles has the benefit of reducing bias in the self-selection of research participants with a particular interest in or positive disposition toward health care technology [21]. As such, the present research study aimed to elicit opinions and experiences from a broad sample of HCPs working in the care of patients with epilepsy, MS or depression.

The Covid-19 pandemic has seen the typical organisation of healthcare in England change to increase remote consultations in both primary [22] and secondary care [23] to reduce the risk of infection between patients and staff. It is therefore timely to explore opinions and experiences of HCPs on the use of smartphone apps and RMT.

## Methods

Surveys are often used to elicit data from large populations and have been used to capture the views of clinicians [24, 25]. Since they require a short amount of time to administer and provide the opportunity to collect both quantitative and qualitative data, a survey was considered to be the most appropriate tool to learn more about HCP experiences, where these individuals have busy schedules and value the ability to complete these at a time of their choosing.

We conducted a large-scale, online survey, administered using the JISC Online Surveys platform [26]. Informed consent was gathered through an information sheet and consent form at the beginning of the survey form. Ethical approval for this study was granted by the University of Nottingham research ethics committee (ref 277-1802) and by the UK Health Research Authority (ref 19/HRA/5041). Inclusion criteria were that respondents should be currently working in the care of people with epilepsy, MS or depression, within adult services.

Our main objectives in the present work were: (i) to determine the extent of smartphone app usage among health care professionals; (ii) to determine the extent of HCP experience of their patients’ use of RMT (including smartphone apps and wearables); and (iii) to understand the impact of patient RMT usage on HCPs’ work, including how this may vary by demographic factors.

There were nine pages of questions in the survey (see Additional file 1), in two broad sections. Textbox 1 shows a breakdown of sections of the survey. Survey questions were designed by the research team to reflect the goals of the research programme. Questions 1–7 concerned participant consent. Questions 8–16 were developed through consultation with expert clinicians from each of the three specialties, together with consideration of existing literature, in order to measure the value of various parameters for target conditions. Questions 17–26 were developed with consideration of potential scenarios of use between patients and clinicians and considering key challenges highlighted by clinical and HCI researchers. Early questions prompted respondents to think about their existing use of apps in their work role before continuing with more focused aspects relevant to making RMT work in clinical practice. The survey was designed pragmatically to elicit data whilst ensuring an accessible survey to encourage participation by busy healthcare professionals.

The survey respondents took a mean of 12 minutes to complete the survey, and were required to do so in a single sitting. In addition to collecting demographic information (questions 3–7), the focus of the first section of the survey was on current and past experience of HCPs’ use of smartphone apps and their patients’ use of RMT (smartphone apps and wearables). The second section had a different focus, exploring the future potential of RMT and the requirements for its future implementation. In this paper, we present results relating only to HCPs’ current/past experience of using smartphone apps and the impact of their patients’ use of RMT on their clinical role.

**Table:** 

**Textbox 1. Survey overview**
SECTION 1:
Page 1: Participant Information and Consent
Page 2: Demographics/Healthcare Professional Role
Page 3: Current use of digital services and devices in your role as a Health Care Professional
SECTION 2:
Page 4: Your thoughts about using digital devices for long term monitoring of medical conditions
Page 5: Value of RMT
Page 6: Accessing and using the data
Page 7: Technical Support requirements
Page 8: Closing remarks

Demographic questions in the survey consisted of respondent age group; specialism; job role; clinical setting; and country of employment. Job role was collected by free text box and during data analysis, participant-provided job roles were sorted into the seven categories reported here. Ambiguous terms were categorised as ‘not specified’.

Question 8 required respondents to indicate whether they had used any kind of smartphone apps in their daily clinical practice (and if so, what genre of app). Question 9 asked respondents to indicate whether they had experienced their patients using different types of RMT devices (smartphone apps, wearable sensing devices or other devices) to improve or increase awareness of their health across six categories of purpose. Question 10 invited respondents to evaluate to what extent the data from patient’s RMT devices had an impact on the respondent’s clinical practice in five areas, each scored on a five-point Likert-style scale. The survey instrument is provided in Additional file 1.

The survey was disseminated via multiple routes:

- The UK National Institute for Health Research (NIHR) Clinical Research Network
- A targeted social media campaign across multiple European countries
- Through the stakeholder group of the Innovative Medicines Institute (IMI)
- Snowballed through professional contacts of the RADAR-CNS consortium members working in five European countries.

The survey was translated from English into five European languages spoken in the countries where the research consortium for the project operated. The first response was received on the 4th February 2019 and the last was received on the 30th March 2020.

### Statistical analysis

Data were analysed using SPSS v.26 and Stata SE 16.1. We used descriptive statistics to understand the rates of responses from different demographic groups. Chi-squared tests were used to compare the rate of reported app usage (question 8) between different job roles and age groups, as well as to compare reported use of apps and devices by respondents’ patients. Responses to items in question 10, on the impact of patient RMT data on five aspects of clinical work, were dichotomised as 1 for responses of ‘definitely’ or ‘sometimes’, and 0 for responses of ‘unsure’, ‘hardly’, and ‘never’. ‘Unsure’ was grouped with the negative polarity items since respondents may have responded in this way if they did not have experience of using these technologies. Dichotomising the data rather than treating it as a 5-point scale had the benefit of resolving this ambiguity. For question 10 on the impact of RMT data on respondents’ clinical practice, univariate, then multivariate logistic regression was used to explore the effect of demographic variables and app usage on views of the impact of RMT data. A *p*-value of 0.05 was considered significant. For each categorical variable, the reference category chosen was the category with the largest number of responses. Missing responses were accounted for using multiple imputation, also conducted in STATA. 83 imputations were calculated for each missing entry, as the highest fraction of missing information (FMI) found in the analysis was 0.83 [27]. A sensitivity analysis was used to examine the influence of missingness on parameter estimates by comparing the consistency of parameters estimates between models with imputed and observed datasets.

## Results

### Recruitment

Survey responses were received from 1009 HCPs. The greatest proportion of these were completed as a result of efforts to recruit via the UK National Institute for Health Research (NIHR) Clinical Research Network: 21 National Health Service (NHS) healthcare trusts and Clinical Commissioning Groups (CCGs) across the UK advertised the survey to healthcare staff working with patients with epilepsy, MS or depression in their area. These trusts and CCGs reported that 650 staff had notified them that they had completed the survey, however it is likely a greater proportion of the total survey responses were obtained via this recruitment method, as it was not compulsory for respondents to notify their trust of their involvement. We did not include a survey item to ask where respondents had heard about the survey to preserve anonymity, so it is not possible to further attribute respondents to different recruitment methods.

Despite the survey being available in 5 European languages in addition to English, and efforts made to disseminate the survey to clinicians working in other countries (via social media, LinkedIn groups, contacts of the consortium, and via the Innovative Medicines Initiative steering group), the number of respondents reporting to be working in countries outside the UK was small (n = 30, 3% of total). These were analysed together with the UK responses as there were too few from each individual country to viably compare them statistically against the UK responses.

### Data cleaning

**Fig. 1:**
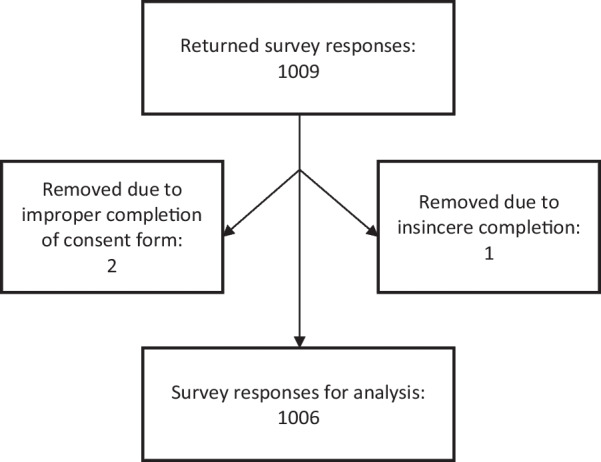
STROBE flow chart showing included responses in the dataset

Of the 1009 completed surveys, 1006 were retained for analysis. Reasons for exclusion were insincere completion of the survey (n = 1) and improper completion of the consent form (n = 2). Figure 1 shows a STROBE flow diagram.

### Demographics

Table 1 shows a breakdown of the included survey responses by demographics. Age group categories followed a normal distribution, with the largest number of respondents in the middle category (ages 41–50, n = 293, 29% of respondents) and progressively fewer in older and younger age groups. The most frequently selected item under *specialism* was ‘mental health’ (n = 587, 38%), with the fewest selecting ‘social care’ (n = 32, 2%). Although relatively few respondents reported a specialism of ‘multiple sclerosis’ (n = 55, 4%) or ‘epilepsy’ (n = 73, 5%), a larger number reported working in neurology (n = 112, 7%), which is a specialism covering both these conditions. Among job roles, ‘nursing’ was the category with the largest proportion of responses. Doctors would have been the second most numerous category, however doctors were split into two categories of ‘GPs’ (n = 118, 12%) and ‘Doctor (excluding GP)’, (n = 138, 14%). ‘Clinical psychology professionals’ were therefore the second most numerous category, with 157 responses (16%). There was good representation from all clinical settings, with fewest (5%) from ‘specialist tertiary care’ settings, which might be expected due to their specialised nature. Most responses (97%) were from HCPs working in the UK. The second most reported country was Portugal, with 21 survey completions (2%).

**Table 1:** Demographics of respondents to the survey

Category	n	%
*Age*		
18–30	161	16.0
31–40	248	24.7
41–50	293	29.1
51–60	247	24.6
60+	52	5.2
No response	5	
*Specialism*		
Neurology	112	11.1
Mood disorders	121	12.0
Mental health	587	58.3
Epilepsy	73	7.3
Multiple sclerosis	55	5.5
Depression	165	16.4
General practice	152	15.1
Psychology	126	12.5
Social care	32	3.2
Other	119	11.8
*Job role*		
Allied health professionals	112	11.1
Doctor (excl GP)	138	13.7
GP	118	11.7
Research/healthcare science	24	2.4
Management	40	4.0
Nursing	268	26.6
Pharmacy	15	1.5
Psychological professions	157	15.6
Student	10	1.0
Wider healthcare team	76	7.6
Not clear	48	4.8
*Clinical setting*		
Primary care/general practice	193	19.2
Secondary care—hospital trust, inpatients	91	9.0
Secondary care—hospital trust, outpatients	82	8.2
Secondary care—mental health trust, inpatients	127	12.6
Secondary care—mental health trust, outpatients	254	25.2
Specialist tertiary care centre	54	5.3
Community care	173	17.2
Other	32	3.2
*Country worked in*		
United Kingdom	974	96.8
Portugal	21	2.1
Belgium	2	0.1
Italy	2	0.1
Germany	1	0.1
Ireland	1	0.1
Israel	1	0.1
Mexico	1	0.1
Switzerland	1	0.1
No response	2	

### Use of smartphone apps by clinicians

**Fig. 2:**
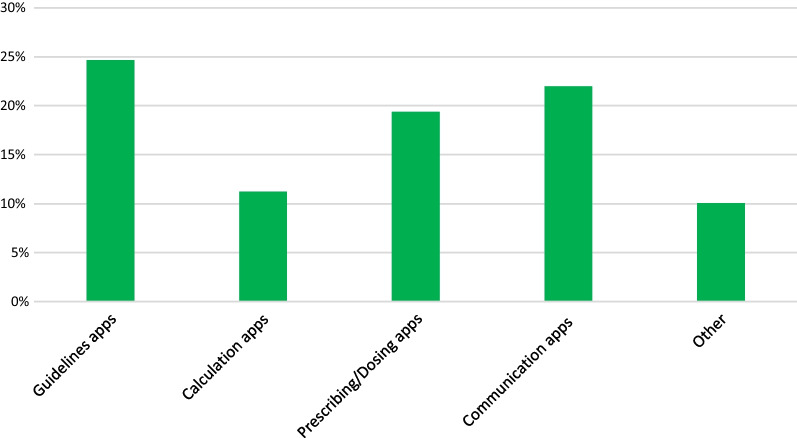
Percentage of survey respondents who report using smartphone apps of each of these types on a daily basis in their professional role. Data were collected in survey question 8: ‘Are there any apps that you currently use on a daily basis in your clinical practice?'

559 respondents (56%) indicated that they use digital health smartphone apps for at least one purpose in their clinical role. Figure 2 shows the percentages of respondents who reported using smartphone apps of each of the five types listed in this question. Guidelines apps were the most used (25%), while calculation apps were least used (11%). There was a spread of responses across all app types, with no app type receiving fewer than 10% of total responses.

**Fig. 3:**
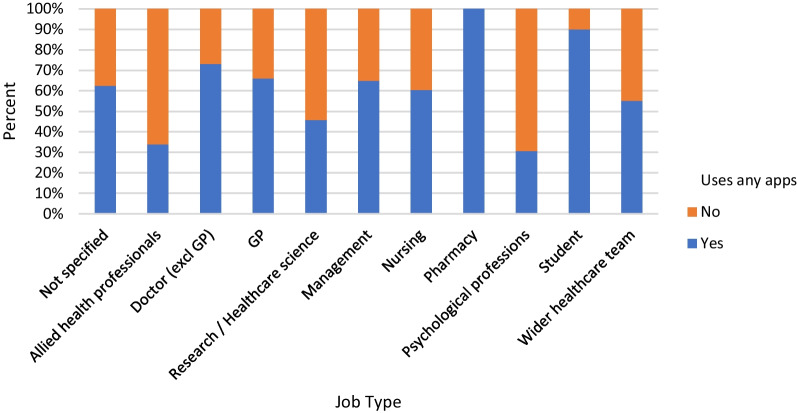
Percentage of respondents in each job category who report using smartphone apps on a daily basis in their clinical practice

Figure 3 shows the percentage of respondents in each job category who reported using smartphone apps in their daily clinical practice. A chi-squared test showed that there were significant differences between job roles for this question (χ^2^ = 106.348, p < 0.01). Pharmacists and medical students were most likely to report using any apps, although there were fewer survey completions overall from these job roles. Further analysis revealed that of the different types of app listed, pharmacists were most likely to report using prescribing/dosing apps (87% of pharmacist respondents), and medical students were most likely to use guidelines apps (80% of student respondents). 40% of GP respondents and 38% of doctors excluding GPs reported using guidelines apps. Clinical psychology professionals and allied health professionals were least likely to report using smartphone apps of any kind.

Question 8a invited respondents to provide examples of other types of app that they used in their daily clinical practice. Table 2 provides a random subsample of the responses, with information on the publisher, category, cost and target condition of these, where relevant. Responses covered apps published by the NHS, charities, commercial organisations, and public bodies. Apps mentioned included a large number targeting mental health, with fewer focussed on neurological conditions, which could be expected given the larger proportion of responses from mental health specialists in the sample. Many of these apps are either patient-facing or have a focus on wellbeing and it is possible that respondents understood this question to mean apps they recommend to their patients, rather than apps they use themselves to facilitate their job role.

**Table 2:** A sample of names and types of smartphone apps reported by respondents who selected ‘other’ in response to a question on types of apps used in their daily clinical practice

App name/genre (as reported)	Category	Target condition (MH/neurology/general)	Publisher	Cost
Survey apps	Communication apps	General	N/A	N/A
3d brain app	Other	Neurology	DNA Learning Centre	Free
British National Formulary	Prescribing/dosing app	General	Indextra AB	$69.99
Consultant-connect	Communication	General	Consultant Connect	Requires subscription by service
Headspace	Consumer	Wellbeing	Headspace (company)	Free, in-app subscription for more content
Calm	Consumer	Wellbeing	Calm (company)	Free, in-app subscription for more content
Medscape	Other	General	WebMD	Free, contains ads
Mindfulness apps	Consumer	Wellbeing	N/A	N/A
Observations apps	Other	General	N/A	N/A
distrACT	Patient-facing	Mental health	Expert Self Care Ltd	Free
Rise Up	Patient-facing	Mental health	Recovery warriors	Free
Rio	Other	General	Servelec	Requires subscription by service
Silvercloud	Communication/patient-facing	Mental health	SilverCloud Health	Free, requires referral
Stay Alive	Patient-facing	Mental health	Grassroots Suicide Prevention	Free
Symptom tracker	Patient-facing	General	N/A	N/A
Wellmind	Patient-facing	Mental health	Blue Step Solutions	Free
Young epilepsy	Patient-facing	Neurology	Young epilepsy charity	No longer available

Across all age categories, between 50 and 60 percent of respondents reported using apps. The highest percentage was reported by those in the over-60 category. However, a chi-squared test on these age groupings was non-significant (χ2 = 1.848, p = 0.76).

### Patient use of RMT for health-related purposes

**Fig. 4:**
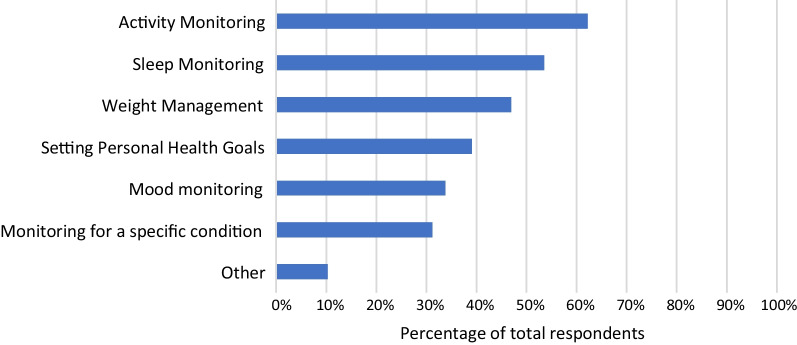
Graph showing percentages of respondents who reported that their patients use smartphone apps, wearable sensing devices or other devices for different purposes

78% of respondents indicated that their patients used RMT (one or more of smartphone apps, wearables, or other type of device) to improve or increase awareness of their health. Figure 4 shows the relevant percentages for different purposes. Activity monitoring was selected by the largest proportion of respondents (62%). Sleep monitoring was also selected by more than half of respondents (53%). Of the given categories, monitoring for a specific condition was least selected (31%).

**Fig. 5:**
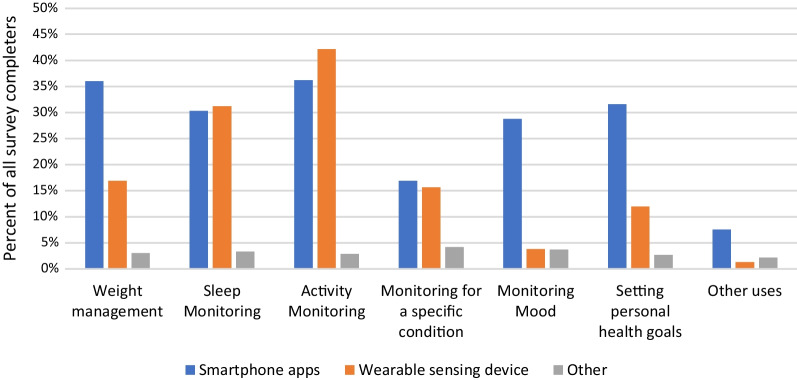
Percentages of survey respondents indicating that their patients use devices of these types for different purposes

Figure 5 shows the split of these responses across different device types. Further to the above, this shows that wearable sensing devices are most commonly reported to be used for passive monitoring of activity and sleep, while smartphone apps were used more often than wearable devices for monitoring mood, setting personal health goals and weight management.

**Fig. 6:**
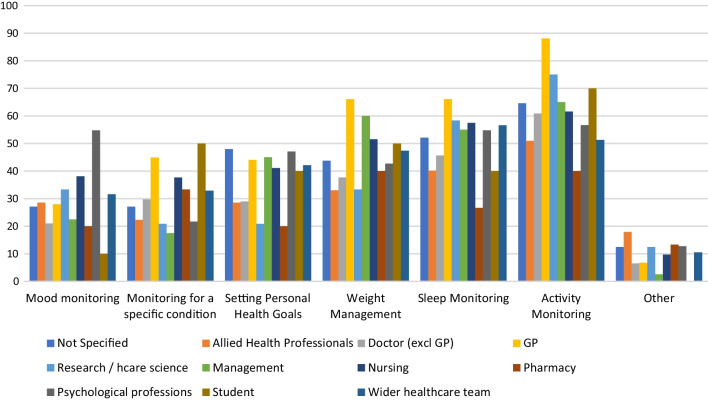
Percentage of each job type indicating they had experience of their patients using RMT for each of these purposes

A sub-group analysis by job role showed that there were significant differences between job roles for this question (χ^2^ = 154.952, p < 0.01). This is shown in Fig. 6. A greater proportion of GPs than other types of health professional had experienced their patients using devices to monitor activity (88% of GP respondents), monitor sleep (66%) and manage weight (66%).

### Impact of RMT data on respondents’ clinical work

**Fig. 7:**
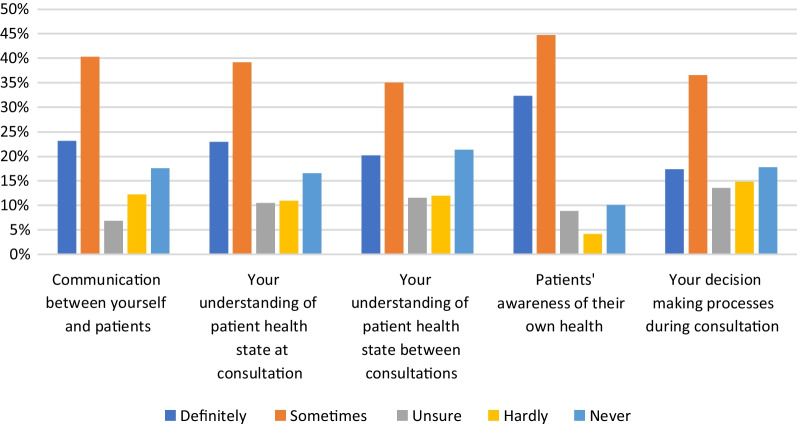
Percentages of item respondents for question items 10.1–10.6 indicating whether RMT data has an impact on five different aspects of their work

Sub-questions 10.1–10.5 asked respondents about the impact of the data from patients’ RMT devices on different aspects of respondents’ clinical work. Figure 7 shows these results. Across all sub-questions, the mean percentage of respondents indicating that the data from RMT ‘definitely’ or ‘sometimes’ had an impact on their work was 62%. The mean percentage of those indicating that it ‘hardly’ or ‘never’ had an impact was 27%. A greater proportion of respondents selected ‘definitely’ or ‘sometimes’ for sub-question 10.4 (Does RMT data impact patients’ awareness of their own health?) than for any of the other sub-questions.

Results from univariate and multivariate logistic regression analyses with multiple imputation and using questions 3–8 as independent variables and sub-questions 10.1–10.5 as dependent variables are provided in Tables 3, 4, 5, 6 and 7. An analysis for multicollinearity revealed no variance inflation factor (VIF) greater than 3.5, suggesting that multicollinearity did not affect the results. Results from multiple imputation and observed datasets were consistent in term of the parameter estimate and its precision.

**Table 3:** Question 10.1—Does data from patients' RMT impact your communication with patients?

Question content/variable/category	Multivariate model	Univariate model
Impact of data on communication with patient	OR (95% CI), p-value	OR (95% CI), p-value
*Q3—age*		
18–30 years	0.694 (0.432, 1.115), 0.1310	0.753 (0.494, 1.150), 0.1900
31–40 years	1.050 (0.697, 1.582), 0.8140	1.045 (0.705, 1.549), 0.8280
41–50 years	Reference category	Reference category
51–60 years	1.167 (0.755, 1.802), 0.4870	1.136 (0.746, 1.729), 0.5510
Over 60 years	1.121 (0.538, 2.336), 0.7600	1.198 (0.596, 2.405), 0.6120
*Q4—specialism*		
Mental health	Reference category	Reference category
Neurology	0.999 (0.643, 1.553), 0.9980	1.031 (0.675, 1.574), 0.8880
Not specified	1.152 (0.789, 1.682), 0.4630	1.163 (0.810, 1.670), 0.4140
*Q5—job role*		
Doctor excluding GP	Reference category	Reference category
Allied health professional	1.356 (0.712, 2.582), 0.3540	0.909 (0.510, 1.621), 0.7470
Not specified	1.269 (0.606, 2.657), 0.5280	1.065 (0.517, 2.194), 0.8640
GP	1.453 (0.624, 3.382), 0.3860	**2.006 (1.125, 3.577), 0.0180**
Research/healthcare scientist	0.592 (0.214, 1.638), 0.3130	0.475 (0.182, 1.240), 0.1280
Management	0.730 (0.322, 1.658), 0.4520	0.685 (0.314, 1.495), 0.3420
Nursing	1.289 (0.776, 2.141), 0.3270	1.055 (0.664, 1.676), 0.8220
Pharmacy	1.364 (0.286, 6.494), 0.6960	1.598 (0.356, 7.184), 0.5400
Clinical psychology professional	1.272 (0.718, 2.256), 0.4100	0.910 (0.548, 1.512), 0.7170
Student	2.822 (0.427, 18.635), 0.2810	1.996 (0.330, 12.062), 0.4510
Wider healthcare team	1.166 (0.571, 2.380), 0.6730	0.956 (0.496, 1.843), 0.8930
*Q6—setting*		
Primary care	Reference category	Reference category
Secondary Care—hospital inpatient	0.688 (0.322, 1.470), 0.3350	0.584 (0.317, 1.073), 0.0830
Secondary care—hospital outpatient	0.750 (0.338, 1.666), 0.4800	0.621 (0.340, 1.133), 0.1210
Secondary care—MH inpatient	0.846 (0.411, 1.742), 0.6500	0.694 (0.407, 1.183), 0.1790
Secondary care—MH outpatient	0.588 (0.323, 1.069), 0.0820	**0.460 (0.299, 0.706), 0.0000**
Specialist tertiary care	1.013 (0.408, 2.515), 0.9780	0.747 (0.363, 1.540), 0.4290
Community care	**0.462 (0.237, 0.900), 0.0230**	**0.414 (0.256, 0.668), 0.0000**
Other	0.505 (0.187, 1.361), 0.1760	**0.400 (0.172, 0.933), 0.0340**
*Q8—Uses apps in daily clinical practice*		
No	Reference category	Reference category
Yes	**1.697 (1.202, 2.398), 0.0030**	**1.727 (1.262, 2.365), 0.0010**

Results from logistic regression with multiple imputation. Multiple imputation was used to impute data for this question item for 301 missing entries out of total 1006. Bold font indicates significant result

**Table 4:** Question 10.2—Does data from patients' RMT impact your understanding of patient health state at consultation?

Question content/variable/category	Multivariate model	Univariate model
Impact of data on understanding of patient health state at consultations	OR (95% CI), p	OR (95% CI), p
*Q3—age*		
18–30 years	0.721 (0.435, 1.196), 0.2050	0.757 (0.480, 1.193), 0.2290
31–40 years	0.857 (0.557, 1.321), 0.4850	0.814 (0.542, 1.225), 0.3240
41–50 years	Reference category	Reference category
51–60 years	1.236 (0.827, 1.847), 0.3020	1.174 (0.799, 1.725), 0.4150
Over 60 years	1.285 (0.596, 2.772), 0.5220	1.307 (0.639, 2.672), 0.4620
*Q4—specialism*		
Mental health	Reference category	Reference category
Neurology	0.720 (0.467, 1.108), 0.1350	0.756 (0.503, 1.137), 0.1790
Not specified	0.983 (0.670, 1.442), 0.9300	1.006 (0.700, 1.446), 0.9750
*Q5—job role*		
Doctor excluding GP	Reference category	Reference category
Allied health professional	0.898 (0.490, 1.647), 0.7280	0.599 (0.343, 1.048), 0.0730
Not specified	1.160 (0.532, 2.526), 0.7090	0.958 (0.449, 2.045), 0.9130
GP	1.051 (0.459, 2.409), 0.9060	1.453 (0.820, 2.574), 0.2000
Research/healthcare scientist	0.460 (0.161, 1.314), 0.1470	**0.340 (0.124, 0.933), 0.0360**
Management	0.624 (0.258, 1.506), 0.2940	0.598 (0.260, 1.378), 0.2270
Nursing	1.282 (0.770, 2.133), 0.3390	1.071 (0.669, 1.715), 0.7740
Pharmacy	1.057 (0.245, 4.565), 0.9410	1.333 (0.327, 5.428), 0.6880
Clinical psychology professional	0.856 (0.471, 1.556), 0.6100	0.637 (0.376, 1.078), 0.0930
Student	2.520 (0.343, 18.501), 0.3630	1.867 (0.277, 12.579), 0.5210
Wider healthcare team	0.950 (0.477, 1.891), 0.8840	0.778 (0.413, 1.466), 0.4370
*Q6—setting*		
Primary care	Reference category	Reference category
Secondary care—hospital inpatient	0.763 (0.347, 1.676), 0.5000	0.732 (0.397, 1.351), 0.3190
Secondary care—hospital outpatient	0.680 (0.321, 1.438), 0.3120	0.658 (0.375, 1.157), 0.1460
Secondary care—MH inpatient	0.754 (0.371, 1.535), 0.4360	0.719 (0.431, 1.201), 0.2070
Secondary care—MH outpatient	0.656 (0.356, 1.209), 0.1760	**0.558 (0.364, 0.856), 0.0070**
Specialist tertiary care	0.858 (0.367, 2.005), 0.7240	0.752 (0.373, 1.514), 0.4240
Community care	**0.471 (0.241, 0.920), 0.0280**	**0.464 (0.288, 0.745), 0.0020**
Other	0.419 (0.153, 1.149), 0.0910	**0.395 (0.166, 0.941), 0.0360**
*Q8—uses apps in daily clinical practice*		
No	Reference category	Reference category
Yes	**1.886 (1.341, 2.654), 0.0000**	**2.010 (1.469, 2.749), 0.0000**

Results from logistic regression with multiple imputation. Multiple imputation was used to impute data for this question item for 301 missing entries out of total 1006. Bold font indicates significant result

**Table 5:** Question 10.3—Does data from patients' RMT impact your understanding of patient health state between consultations?

Question content/variable/category	Multivariate model	Univariate model
Impact of data on understanding of patient health state between consultations	OR (95% CI), p	OR (95% CI), p
*Q3—age*		
18–30 years	1.088 (0.647, 1.830), 0.7490	1.097 (0.680, 1.770), 0.7040
31–40 years	1.244 (0.821, 1.884), 0.3030	1.233 (0.827, 1.838), 0.3030
41–50 years	Reference category	Reference category
51–60 years	1.116 (0.766, 1.627), 0.5670	1.101 (0.766, 1.584), 0.6030
Over 60 years	1.433 (0.675, 3.038), 0.3480	1.553 (0.764, 3.158), 0.2240
*Q4—specialism*		
Mental health	Reference category	Reference category
Neurology	1.077 (0.707, 1.641), 0.7280	1.105 (0.740, 1.651), 0.6240
Not specified	1.421 (0.973, 2.074), 0.0690	1.411 (0.979, 2.035), 0.0650
*Q5—job role*		
Doctor excluding GP	Reference category	Reference category
Allied health professional	1.063 (0.561, 2.016), 0.8510	0.766 (0.427, 1.376), 0.3720
Not specified	1.535 (0.734, 3.207), 0.2550	1.267 (0.620, 2.591), 0.5160
GP	1.775 (0.813, 3.875), 0.1490	**1.786 (1.041, 3.064), 0.0350**
Research/healthcare scientist	0.912 (0.340, 2.448), 0.8560	0.805 (0.315, 2.057), 0.6510
Management	0.725 (0.305, 1.724), 0.4660	0.641 (0.278, 1.475), 0.2950
Nursing	1.358 (0.827, 2.231), 0.2270	1.122 (0.710, 1.771), 0.6220
Pharmacy	1.633 (0.380, 7.027), 0.5090	1.755 (0.431, 7.140), 0.4320
Clinical psychology professional	1.374 (0.762, 2.478), 0.2900	1.012 (0.605, 1.693), 0.9640
Student	1.235 (0.251, 6.070), 0.7950	1.285 (0.278, 5.946), 0.7480
Wider healthcare team	1.395 (0.717, 2.716), 0.3270	1.138 (0.616, 2.105), 0.6790
*Q6—setting*		
Primary care	Reference category	Reference category
Secondary care—hospital inpatient	1.037 (0.495, 2.173), 0.9240	0.788 (0.441, 1.408), 0.4220
Secondary care—hospital outpatient	0.891 (0.422, 1.880), 0.7610	0.678 (0.384, 1.199), 0.1820
Secondary care—MH inpatient	0.954 (0.482, 1.885), 0.8910	0.767 (0.461, 1.277), 0.3080
Secondary care—MH outpatient	0.860 (0.478, 1.547), 0.6150	**0.613 (0.401, 0.937), 0.0240**
Specialist tertiary care	1.424 (0.626, 3.237), 0.3990	0.941 (0.483, 1.833), 0.8570
Community care	0.725 (0.385, 1.366), 0.3190	**0.551 (0.348, 0.871), 0.0110**
Other	0.514 (0.175, 1.511), 0.2260	**0.370 (0.144, 0.951), 0.0390**
*Q8—uses apps in daily clinical practice*		
No	Reference category	Reference category
Yes	**1.760 (1.265, 2.449), 0.0010**	**1.781 (1.311, 2.418), 0.0000**

Results from logistic regression with multiple imputation. Multiple imputation was used to impute data for this question item for 303 missing entries out of total 1006. Bold font indicates significant result

**Table 6:** Question 10.4—Does data from patients' RMT impact patients' awareness of their own health?

Question content/variable/category	Multivariate model	Univariate model
Impact of data on patients' awareness of their own health	OR (95% CI), p	OR (95% CI), p
*Q3—age*		
18–30 years	0.861 (0.502, 1.476), 0.5850	0.914 (0.569, 1.467), 0.7080
31–40 years	1.237 (0.790, 1.937), 0.3530	1.204 (0.790, 1.837), 0.3880
41–50 years	Reference category	Reference category
51–60 years	1.147 (0.752, 1.750), 0.5250	1.113 (0.745, 1.660), 0.6010
Over 60 years	1.423 (0.591, 3.426), 0.4310	1.450 (0.645, 3.262), 0.3680
*Q4—specialism*		
Mental health	Reference category	Reference category
Neurology	0.805 (0.500, 1.297), 0.3720	0.831 (0.531, 1.298), 0.4150
Not specified	0.910 (0.609, 1.360), 0.6460	0.958 (0.656, 1.398), 0.8240
*Q5—job role*		
Doctor excluding GP	Reference category	Reference category
Allied health professional	1.102 (0.566, 2.143), 0.7750	0.814 (0.448, 1.476), 0.4970
Not specified	0.955 (0.432, 2.112), 0.9090	0.835 (0.391, 1.783), 0.6410
GP	0.834 (0.297, 2.345), 0.7310	**2.501 (1.280, 4.887), 0.0070**
Research/healthcare scientist	0.859 (0.306, 2.408), 0.7720	0.688 (0.257, 1.840), 0.4560
Management	0.645 (0.279, 1.489), 0.3040	0.587 (0.262, 1.312), 0.1940
Nursing	1.089 (0.640, 1.854), 0.7520	0.994 (0.610, 1.620), 0.9820
Pharmacy	0.429 (0.108, 1.700), 0.2280	0.457 (0.125, 1.665), 0.2350
Clinical psychology professional	1.744 (0.884, 3.440), 0.1090	1.558 (0.838, 2.899), 0.1610
Student	1.507 (0.272, 8.346), 0.6380	1.023 (0.197, 5.308), 0.9780
Wider healthcare team	0.771 (0.378, 1.574), 0.4740	0.649 (0.339, 1.242), 0.1920
*Q6—setting*		
Primary care	Reference category	Reference category
Secondary care—hospital inpatient	**0.252 (0.101, 0.627), 0.0030**	**0.251 (0.130, 0.486), 0.0000**
Secondary care—hospital outpatient	0.403 (0.153, 1.063), 0.0660	**0.422 (0.207, 0.859), 0.0170**
Secondary care—MH inpatient	**0.272 (0.118, 0.631), 0.0020**	**0.261 (0.146, 0.465), 0.0000**
Secondary Care—MH outpatient	**0.291 (0.130, 0.651), 0.0030**	**0.309 (0.184, 0.517), 0.0000**
Specialist tertiary care	0.414 (0.140, 1.217), 0.1090	0.437 (0.191, 1.002), 0.0500
Community care	**0.240 (0.103, 0.559), 0.0010**	**0.251 (0.142, 0.442), 0.0000**
Other	**0.222 (0.069, 0.709), 0.0110**	**0.210 (0.084, 0.525), 0.0010**
*Q8—uses apps in daily clinical practice*		
No	Reference category	Reference category
Yes	**1.678 (1.178, 2.390), 0.0040**	**1.496 (1.091, 2.051), 0.0120**

Results from logistic regression with multiple imputation. Multiple imputation was used to impute data for this question item for 282 missing entries out of total 1006. Bold font indicates significant result

**Table 7:** Question 10.5—Does data from patients' RMT impact your decision-making processes during consultation?

Question content/variable/category	Multivariate model	Univariate model
Impact of data on decision-making during consultation	OR (95% CI), p	OR (95% CI), p
*Q3—age*		
18–30 years	**0.593 (0.358, 0.983), 0.0430**	**0.610 (0.382, 0.976), 0.0390**
31–40 years	0.724 (0.474, 1.105), 0.1340	0.695 (0.461, 1.048), 0.0830
41–50 years	Reference category	Reference category
51–60 years	0.835 (0.555, 1.257), 0.3870	0.816 (0.548, 1.214), 0.3150
Over 60 years	0.696 (0.334, 1.448), 0.3310	0.767 (0.383, 1.533), 0.4520
*Q4—specialism*		
Mental health	Reference category	Reference category
Neurology	0.784 (0.514, 1.197), 0.2600	0.779 (0.520, 1.168), 0.2270
Not specified	0.990 (0.695, 1.411), 0.9560	0.977 (0.696, 1.371), 0.8920
*Q5—job role*		
Doctor excluding GP	Reference category	Reference category
Allied health professional	1.014 (0.552, 1.865), 0.9640	0.736 (0.419, 1.292), 0.2850
Not specified	0.948 (0.438, 2.053), 0.8930	0.806 (0.385, 1.688), 0.5670
GP	0.494 (0.221, 1.101), 0.0850	0.904 (0.519, 1.574), 0.7210
Research/healthcare scientist	0.504 (0.169, 1.506), 0.2200	0.382 (0.133, 1.097), 0.0740
Management	0.661 (0.273, 1.602), 0.3590	0.620 (0.268, 1.431), 0.2620
Nursing	1.090 (0.648, 1.834), 0.7450	0.938 (0.580, 1.515), 0.7920
Pharmacy	0.975 (0.247, 3.856), 0.9720	1.182 (0.314, 4.457), 0.8040
Clinical psychology professional	0.772 (0.414, 1.439), 0.4140	0.627 (0.362, 1.086), 0.0960
Student	0.789 (0.133, 4.662), 0.7930	0.521 (0.098, 2.776), 0.4450
Wider healthcare team	0.854 (0.437, 1.670), 0.6450	0.689 (0.367, 1.293), 0.2460
*Q6—setting*		
Primary care	Reference category	Reference category
Secondary care—hospital inpatient	0.485 (0.226, 1.040), 0.0630	0.703 (0.392, 1.260), 0.2360
Secondary care—hospital outpatient	0.532 (0.247, 1.146), 0.1070	0.830 (0.468, 1.473), 0.5240
Secondary care—MH inpatient	0.571 (0.287, 1.136), 0.1100	0.823 (0.498, 1.359), 0.4470
Secondary care—MH outpatient	**0.426 (0.236, 0.769), 0.0050**	**0.576 (0.376, 0.882), 0.0110**
Specialist tertiary care	0.885 (0.369, 2.120), 0.7830	1.233 (0.606, 2.510), 0.5630
Community care	**0.415 (0.219, 0.789), 0.0070**	**0.629 (0.398, 0.994), 0.0470**
Other	**0.266 (0.091, 0.782), 0.0160**	**0.384 (0.150, 0.982), 0.0460**
*Q8—uses apps in daily clinical practice*		
No	Reference category	Reference category
Yes	**1.600 (1.129, 2.268), 0.0080**	**1.666 (1.195, 2.323), 0.0030**

Results from logistic regression with multiple imputation. Multiple imputation was used to impute data for this question item for 306 missing entries out of total 1006. Bold font indicates significant result

Across all five sub-questions in question 10, the independent variable on HCP use of smartphone apps (question 8), gave a significant result in the multivariate logistic regression models, indicating that those respondents who reported using apps were more likely to respond with ‘definitely’ or ‘sometimes’ to the items discussing the impact of data from RMT on their work.

In relation to clinical setting, the reference category across all regression analyses in sub-questions 10.1 to 10.5 was ‘primary care’. The univariate analyses showed that in all 5 sub-questions, respondents from at least one category of setting gave responses which were significantly different to those in primary care. The odds ratios in these cases were below one, indicating that respondents working in multiple settings outside of primary care were less likely than those in primary care to respond positively (choose ‘definitely’ or ‘sometimes’ rather than ‘unsure’, ‘hardly’ or ‘never’) concerning the impact of RMT on their work. However, in the multivariate regression analyses, only 4 out of 5 sub-questions (10.1, 10.2, 10.4 and 10.5) showed significant differences between primary care and other settings, when other variables were controlled for.

With regard to job role, the reference category was ‘doctors (excluding GPs)’. In 3 out of the 5 question items, the general practitioner job role was significantly different to the reference category (doctors excluding GPs) in the univariate regression analysis, however this was not sustained in the multivariate models for these sub-questions where other variables were controlled for.

In relation to age, only sub-question 10.5, concerning the impact of RMT data on decision-making processes in consultations, showed any difference between age groups. Here, according to both univariate and multivariate models, respondents in the 18–30 age category were less likely than those in the reference category (ages 41–50) to indicate that patients’ RMT data had an impact on their decision-making processes during consultation. There was no significant difference between any specialism and the reference category (mental health) in the regression models for any of the question items.

## Discussion

This study achieved its aim of eliciting views from a large sample of healthcare staff working in the care of patients with epilepsy, MS or depression. The results from this study have added to prior work in this area [19, 20] by showing that smartphone apps are used by more than half (56%) of HCPs and that more than three quarters (78%) report that their patients use RMTs for health-related purposes. These figures are greater than in previous research studies that surveyed the use of apps in treatment by healthcare professionals working in epilepsy, where 45% of respondents previously reported using apps with their patients [28], and in depression, where 21.2% of respondents in a prior study stated they used apps in treatment for depression [25]. Our findings thus may indicate their growing use in clinical practice (no prior evidence is available on clinicians’ use of apps in MS).

HCPs reported that their patients use smartphone apps for the purposes of: managing weight; setting personal health goals; and monitoring sleep, activity and mood. Wearable sensing devices are used by their patients for sleep and activity monitoring, and a smaller percentage report patient use of these devices for weight management and monitoring a specific condition (Fig. 5). The majority of HCPs (mean average 62%) indicated that the data from these RMTs impacts their clinical practice and also indicated that RMT data has an impact on their patients’ awareness of their own health (see Fig. 7). Specifically, RMT data was found to impact on communication with patients, on HCPs’ understanding of patient health state, and on HCPs’ decision-making processes. Regression analyses show that HCP views on the impact of RMT data vary according to whether respondents use apps themselves. This adds to recent research in the area of depression care indicating an association between level of technology experience and consideration of use of apps in clinical practice [25]. Views on the impact of RMT data were also found to vary according to clinical setting, job role and age.

Our results suggest RMT is currently having a greater impact in primary care than in other healthcare sectors. A higher proportion of general practitioner respondents reported their patients used devices to monitor activity and sleep and manage weight than in other job roles. This suggests a greater awareness among GPs than among other health care professionals concerning the devices patients use, and the purposes for which they may use them. In addition, GPs were significantly more likely than other kinds of doctor to indicate that RMT data had an impact on their work, on 3 out of 5 questions on this topic (see Tables 3, 4, 5, 6 and 7). Furthermore, those working in primary care were significantly more likely than those in secondary care (mental health outpatient), community care and ‘other’ settings to report that RMT data impacted their work, on all five questions on this topic. This may be because patients are more likely to discuss the data from devices they have bought themselves with primary care practitioners, who they see more regularly, and for whom relational aspects of their work are considered more important. While prior work in both Denmark [29] and the UK [20] has shown that general practitioners recognise benefits to patient use of wearables and apps, the present study has demonstrated more precisely how the attitudes of primary care professionals differ from those in other areas of care.

Our sub-group analysis of job roles that use apps in their clinical practice revealed that a larger proportion of pharmacists (100%) and medical students (90%) use apps in their clinical practice than those in other job categories. The most commonly used app types by these groups were prescribing/dosing apps and guidelines apps, respectively. However, it is important to note that these job roles were only represented by a small number of respondents relative to other job roles (15 pharmacists and 10 medical students), meaning it is possible that only those very most interested in the application of technology chose to take part in this study.

Our data on the type of RMT device used by patients for each health-related purpose (Fig. 6) showed that use of wearables was less prevalent than use of smartphones for certain purposes (weight management, setting personal health goals, monitoring mood). Recalling that this survey was conducted with HCPs rather than patients, it is possible that HCPs are unaware that their patients use wearables in these ways where patients do not deem it relevant to inform clinicians of this, and thus their use may be under-represented in these survey results. Wearables are not as ubiquitous as smartphones, and this may also explain the lower reported use of these for health-related purposes. Another possible explanation is that wearable devices have smaller screens and lack the space to incorporate an easy method for manual data entry and are thus better suited to ‘passive’ means of data collection than ‘active’ means.

In comparison to other health conditions, this survey, targeting HCPs working in the care of people with MS, epilepsy or depression, demonstrated a higher proportion of respondents reporting patients using RMT for health related purposes (78%) than was the case in studies on diabetes (type 1: < 60%, type 2: ~ 33%) [1] and hypertension (< 50%) [2]. Given the recency of these prior studies, it is unlikely that this is simply a result of technologies becoming more widespread, and may reflect a difference in how these conditions are managed, with both blood pressure and blood sugar being measured using bespoke (non-wearable) devices, and therefore not requiring a smartphone app to be able to measure the symptoms of these conditions.

Our work is novel in exploring views on the application of these technologies in the care of people with epilepsy, MS, or depression, from a large sample. This evidence adds to a growing body of work understanding the relationships between healthcare technology use, competence and adoption by healthcare professionals. Recent work by Vallo et al. [30] indicates that adoption and use of digital technology are “related to perceived performance, social influence and organisational context” and the role of ICT [Information and Communication Technology] in clinical professional development. This work highlights the need for a holistic service design approach [31] to reduce external and internal barriers to adoption and optimise the benefits and professional competence of use of these technologies in practice [32].

### Recommendations for future work

While HCPs reported using apps, some of the app names they reported appear to be patient-facing, and it is not clear from our data whether they are using these apps themselves or are recommending these to their patients. We cannot tell from our study the extent to which apps and wearables are prescribed or recommended to patients by healthcare professionals. In the UK, some smartphone apps are now highlighted in the NHS apps library [33], and the National Institute for Health and Care Excellence (NICE) has issued guidance that HCPs consider digital mobile health interventions, including smartphone apps, for behaviour change [34]. However, the present study has not been able to ascertain how many HCPs actively recommend these to their patients and this is an interesting question for future work. In other European countries including Germany and Sweden, governments incentivise HCPs to recommend digital solutions to their patients [35]. Further work could also usefully compare use of treatment pathways involving apps and wearables in countries with different incentivisation policies to understand the benefits of these approaches. There are also questions around the regulation of apps and the effect of regulation on their adoption that could be usefully explored.

Recently, NHS guidance has been aimed at primary and secondary care services reducing face-to-face appointments during the COVID-19 pandemic [23, 36] and there is some suggestion that healthcare professionals may have become more favourably disposed to the use of digital technologies to support their clinical practice during this period [37]. However, this evidence relates largely to the use of video conferencing software and telephone consultations rather than the use of wearables and apps to generate data on behavioural and physiological aspects of health, although these are beginning to be evaluated in practice. Given that clinicians would be required to review the data from such RMT on a digital device (e.g. computer) even if patients were present at a face-to-face appointment, it is not clear whether the recent reduction in face-to-face appointments would cause a change of attitudes towards the use of this data in practice. Further work would be required to understand precisely how views on these specific areas may have changed or not because of the reorganisation of care for non-COVID-19 patients during the pandemic.

### Strengths and limitations

A key strength of this study is the large sample size, with over 1000 respondents. Another is the exploration of the impact that RMT data has on patient care. The application of regression techniques has enabled us to control for demographic factors when exploring the variation in these views. Despite a coordinated effort to facilitate responses from clinicians working outside of the UK (translation into multiple European languages, advertisement via social media, network connections), the proportion of those working in other countries among respondents to the survey was small. However, we were able to reach a representative sample of respondents from different age groups, job roles and clinical settings, which has enabled sub-group analyses of the views and experiences presented. There is some risk that the self-selecting sample for this study were those most interested in the use of technology and this may mean that views expressed are more positive than is the case across all healthcare professionals. A small percentage of respondents (6%) were from management or healthcare science roles where their contact with patients, and therefore their understanding of factors relevant to the application of RMT, may be more limited. As an exploratory study, the data were analysed without multiplicity adjustment and results were mainly interpreted for exploratory and not confirmatory purposes [38].

## Conclusions

This survey study achieved its aim of eliciting opinions and experiences from a large sample of HCPs working in the care of patients with epilepsy, MS or depression. Our main objectives in the present work were: (i) to determine the extent of smartphone app usage among health care professionals; (ii) to determine the extent of HCP experience of managing patients’ use of RMT; and (iii) to understand the impact of patient RMT usage on HCPs’ work, including how this may vary by demographic factors. The results show that prior to the COVID-19 pandemic, smartphone apps were already widely used by more than half of HCPs (objective i), and RMT was used by patients of more than three quarters of respondents (objective ii). HCPs widely believed that the data from RMT impacted their clinical practice, although there is some evidence that their views vary according to demographic and technology usage factors (objective iii). We consider it unlikely that views on the usefulness of the data from RMT will be substantially different after the pandemic, given similar practical requirements for its application in both face-to-face and remote consultations, although further research would be needed to test this hypothesis. We do expect that more HCPs will become more familiar with RMT and that adoption of mobile platforms will become more prevalent in routine health and social care settings in the aftermath of COVID-19.

### Supplementary Information

**Additional file 1.** Survey instrument used in the study, including participant consent form.

## Data Availability

The datasets used and/or analysed during the current study are available from the corresponding author on reasonable request.

## Abbreviations

COVID-19: Coronavirus Disease 2019
CCG: Clinical Commissioning Group
FMI: Fraction of Missing Information
ICT: Information Communication Technology
GP: General Practitioner
GPS: Global Positioning System
HCP: Healthcare Professional
MS: Multiple Sclerosis
NHS: National Health Service
NICE: National Institute for Health and Care Excellence
NIHR: National Institute for Health Research
RADAR-CNS: Remote Assessment of Disease and Relapse – Central Nervous System
RMT: Remote Measurement Technology
STROBE: Strengthening the Reporting of Observational Studies in Epidemiology
UK: United Kingdom
