# Correction: The impact of data from remote measurement technology on the clinical practice of healthcare professionals in depression, epilepsy and multiple sclerosis: survey

**DOI:** 10.1186/s12911-023-02390-2

**Published:** 2023-12-11

**Authors:** J. A. Andrews, M. P. Craven, A. R. Lang, B. Guo, R. Morriss, C. Hollis, The RADAR-CNS Consortium

**Affiliations:** 1 NIHR MindTech MedTech Co-operative, Institute of Mental Health, University of Nottingham, Triumph Road, Jubilee Campus, Nottingham, NG7 2TU UK; 2 Mental Health and Clinical Neurosciences, School of Medicine, University of Nottingham, Nottingham, UK; 3 Human Factors Research Group, Faculty of Engineering, University of Nottingham, Nottingham, UK; 4 NIHR Nottingham Biomedical Research Centre, University of Nottingham, Nottingham, UK; 5 ARC-EM, School of Medicine, University of Nottingham, Nottingham, UK; 6 Kings College London, London, UK

**Correction: Andrews et al. BMC Med Inform Decis Mak (2021) 21:282**

10.1186/s12911-021-01640-5

After the publication of the original article [1], the authors reported errors in two statements. The Abstract results section reads: “more than three quarters of their patients use smartphone apps or wearable devices for health-related purposes.” Instead, it should have read: “more than three quarters report that their patients use smartphone apps or wearable devices for health-related purposes.”

Furthermore, the Conclusions section reads: “RMT was used by more than three quarters of their patients”. The statement should have read: “RMT was used by patients of more than three quarters of respondents”.

The original article [1] has been updated.
