# Supplementary material for: The impact of data from remote measurement technology on the clinical practice of healthcare professionals in depression, epilepsy and multiple sclerosis: survey
Source: BMC Med Inform Decis Mak. 2021 Oct 13;21:282. doi: 10.1186/s12911-021-01640-5 (PMC8513566; doi:10.1186/s12911-021-01640-5)
Supplement: Supplementary file 1 — Additional file 1. Survey instrument used in the study, including participant consent form. [file 12911_2021_1640_MOESM1_ESM.pdf]

RADAR-CNS – Use of remote measuring technologies – epilepsy, multiple sclerosis, depression – healthcare professional perspectives and experiences of RMT

Page 1: Participant Information and Consent

RADAR-CNS (www.radar-cns.org) is investigating the use of remote measuring technologies (RMT) in provision of health and social care. We are specifically investigating their use in the care of people with one or more of the following conditions

- epilepsy

- multiple sclerosis (MS)

- depression

We are examining how commercially available lifestyle devices, like FitBits, can be used to monitor and predict changes in a range of clinically relevant factors e.g. in activity, mobility, mood, seizure frequency. The information collected by the devices could be useful for patients and their healthcare team and may help find ways of improving treatment.

This survey investigates healthcare professional views and experiences of RMT and mHealth more generally. This will contribute to our understanding of how these technologies impact clinical pathways and decision points from the perspective of the healthcare professionals.

If you are a healthcare professional providing care for people living with epilepsy/ MS/depression, we would like to have your thoughts about these devices.

We would be grateful if you would complete this questionnaire. We are just looking for your opinions, we are not asking you to take part in any trials.

The survey should take approx 10min of your time.

As a thank you for your time, we will donate £1 to a relevant charity for

every complete questionnaire we receive. At the end of the questionnaire you will be provided with the choice of three charities to donate to.

Thank you very much for your help!

1. Do you agree with the following statements?

	Consent process
	Yes
I understand that all information from this survey will be reported anonymously.	◌
I understand that my data will only be accessed by the research team and will be used cumulatively in any publications and reports.	◌
I understand that my words may be used in reports and publications.	◌
I understand that the survey data will be stored securely in accordance with the University of Nottingham data storage policy (as per the Data Protection Act 2018).	◌
I understand that I can withdraw from this survey at any point until submission.	◌
I am aged 18 years or over.	◌

2. I consent to my participation in this survey and the use of my data in RADAR-CNS outputs. ✱ *Required*

- ◌ Yes
- ◌ No

Page 2: Healthcare Professional role

3. How old are you?

- 18 - 30 years
- 31 - 40 years
- 41 - 50 years
- 51- 60 years
- Over 60 years

4. What is your specialism? (please tick all that apply)

- Neurology
- Mood Disorders
- Mental Health
- Epilepsy
- Multiple Sclerosis
- Depression
- General Practice
- Psychology
- Social Care
- Other

4.a. If you selected Other, please specify:

5. What is the title of your job role? (Please indicate if you have any specialisation e.g. paediatrics, geriatrics etc)

6. What kind of clinical setting do you work in? (If you work in multiple settings please select the one in which you provide most care and spend the most time) ✱ *Required*

- Primary Care / General Practice
- Secondary Care - hospital trust, inpatients
- Secondary Care - hospital trust, outpatients
- Secondary Care- mental health trust, inpatients
- Secondary Care- mental health trust, outpatients
- Specialist Tertiary care centre
- Community Care
- Other

7. What country do you work in?

Page 3: Current use of digital services and devices in your role as a Health Care Professional (HCP)

8. Are there any apps that you currently use on a daily basis in your clinical practice?

- ☐ Guidelines apps
- ☐ Calculation apps
- ☐ Prescribing / dosing apps
- ☐ Communication apps
- ☐ Other

8.a. If you selected Other, please specify:

9. Have you experienced your patients using any of these devices for improving or increasing awareness of their health?

	Device			
	Smartphone apps	Wearable sensing device	Other	If you selected Other, please specify:
Weight Management	☐	☐	☐	
Sleep Monitoring	☐	☐	☐	
Activity Monitoring	☐	☐	☐	
Monitoring for a specific condition	☐	☐	☐	

Monitoring Mood	☐	☐	☐	
Setting personal health goals	☐	☐	☐	
Other	☐	☐	☐	

10. If the data from these technologies gets used in your consultation, please indicate if the data impacts the following factors.

	Does the data impact the following....				
	Definitely	Sometimes	Unsure	Hardly	Never
Communication between yourself and patients	○	○	○	○	○
Your understanding of patient health state at consultation	○	○	○	○	○
Your understanding of patient health state between consultations	○	○	○	○	○
Patients awareness of their own health	○	○	○	○	○
Your decision making processes during consultation	○	○	○	○	○

Page 4: Your thoughts about using digital devices for long term monitoring of medical conditions

11. Which of the following data sets do you think would be useful to be monitored over a period of time? Please answer the column that best describes the area you work in.

	Epilepsy		Multiple Sclerosis		Depression	
	Yes	No	Yes	No	Yes	No
Body movements	○	○	○	○	○	○
Continuous tracking of location using GPS	○	○	○	○	○	○
Heart Rate	○	○	○	○	○	○
Breathing Rate	○	○	○	○	○	○
Smartphone usage	○	○	○	○	○	○
Voice Quality	○	○	○	○	○	○
Sweating	○	○	○	○	○	○
Skin Temperature	○	○	○	○	○	○
Environment features e.g. light, temperature	○	○	○	○	○	○
Sleep Quality	○	○	○	○	○	○

11.a. In your experience and opinion is there any other information / data sets that you think could be useful to track?

12. Do you think it could be useful to collect information on mood, concentration, attention and memory as supplementary data in addition to physiological monitoring?

	Is this data useful?		
	Yes	Unsure	No

Mood	◯	◯	◯
Concentration	◯	◯	◯
Attention	◯	◯	◯
Memory	◯	◯	◯

13. How often do you think it would be useful for the patient to respond to mood based questions?

◯ Daily
◯ Weekly
◯ Monthly
◯ Never
◯ Retrospective responses following medical event e.g. seizure, relapse etc
◯ Other

13.a. If you selected Other, please specify:

Page 5: Value of RMT

14. Who in your organisation / clinical teams do you think would make most use of the data from RMT enabled devices? e.g. would it be yourself, nursing or medical staff, primary / secondary care providers?

15. Do you think your healthcare organisation would benefit from the use of these technologies as part of patients' care plans?

- Highly likely to benefit
- Likely to benefit
- Unsure
- Unlikely to benefit
- Highly unlikely to benefit

15.a. If there is benefit to be experienced, who (other than the patient) is likely to benefit most from the data which is recorded from RMT?

16. Are there any specific decision points in your clinical practices / provision of care where you feel information from monitoring technologies might be useful?

More info

	Yes	Maybe	No
Decision points that rely on specific relapse / seizure measures?	☐	☐	☐

Decision points that rely on other physiological measures that are non specific to relapse?	☐	☐	☐
Decision points that rely on mood based variables?	☐	☐	☐
Decision points that rely on contextual factors?	☐	☐	☐

Page 6: Accessing and using the data

17. How likely are you to access data from the RMT in the following situations?

	Likelihood of access					
	Highly likely	Likely	Maybe	Unlikely	Highly unlikely	Never
In preparation for a consultation with a patient	○	○	○	○	○	○
During a consultation with a patient	○	○	○	○	○	○
In between consultations with a patient if the system were to flag up data/reasons for concern?	○	○	○	○	○	○
Post medical event e.g. seizure and/or relapse	○	○	○	○	○	○

17.a. Are there any other times when you think having access to this kind of data might be useful? Please explain.

18. How would you prefer to gain access to the information? Please rank 1-4 with 1 being the most preferable option and 4 being the least preferable.

Please don't select more than 1 answer(s) per row.

	1	2	3	4
Information made available during consultation via the patient's device (smartphone, tablet etc)	☐	☐	☐	☐

Information made available to you automatically, at any time via a secure portal.	☐	☐	☐	☐
Information made available to you remotely, at the patients discretion.	☐	☐	☐	☐
Information made available via notification if the system detects a downturn in patient health state or potential relapse.	☐	☐	☐	☐

19. For you as a healthcare provider please rank the following statements in order of what aspect of RMT may provide benefit to you in your clinical practice. 1 indicates most benefit and 5 indicates least benefit.

Please don't select more than 1 answer(s) per row.

	1	2	3	4	5
Information about patient health state over a long period of time	☐	☐	☐	☐	☐
Information about patient health state before or in the run up to a medical 'event' e.g. seizure or relapse	☐	☐	☐	☐	☐
Information about patient health state during a medical 'event' e.g. seizure or relapse	☐	☐	☐	☐	☐
Information about specific physiological measures over a long period of time	☐	☐	☐	☐	☐

Information about mental health/mood measures over a long period of time	☐	☐	☐	☐	☐

19.a. Are there any other benefits that you might like to suggest?

20. For you as a healthcare provider, how do you perceive the potential challenges associated with RMT use? Please rank the following questions in order of concern, where 1 indicates you are most concerned and 5 indicates you are least concerned.

Please don't select more than 1 answer(s) per row.

	1	2	3	4	5
Additional workload for yourself	☐	☐	☐	☐	☐
Burden of having too much information	☐	☐	☐	☐	☐
Time to review information	☐	☐	☐	☐	☐
Adherence of patients to using the technology	☐	☐	☐	☐	☐
Patient anxiety related to health monitoring and access to data	☐	☐	☐	☐	☐

20.a. Are there any other challenges you might like to suggest?

21. If a patient were to use this technology in monitoring their health condition, how do you think this would impact on your job? Please respond to each of the individual following statements with one answer

	How much do you agree with each of these statements?				
	Strongly Agree	Agree	Neutral	Disagree	Strongly Disagree
RMTs will help in my management of patients	○	○	○	○	○
You can have too much information about patients health state	○	○	○	○	○
I think that these technologies provide more value to patients than clinicians	○	○	○	○	○
I like to engage with novel technologies in my work	○	○	○	○	○
I think technology is a good tool for helping patients manage their condition themselves	○	○	○	○	○
RMT could fit well into my clinical practice	○	○	○	○	○
RMT could over complicate the healthcare system	○	○	○	○	○
If RMT was proven to detect relapse, it then has the potential to 'free up' resources	○	○	○	○	○

Page 7: Technical Support Requirements

22. How much technical support do you think you would need to be able to make use of the data being collected by the device? Please select one.

- None, I would work it out for myself
- A one off training session
- Optional daily support
- Optional weekly support
- Ad hoc support

23. Please rank your preference regarding mode of training/technical support from 1 (most preferred) to 6 (least preferred).

Please don't select more than 1 answer(s) per row.

	1	2	3	4	5	6
Person to person	☐	☐	☐	☐	☐	☐
Document based instructions for use (IFU)	☐	☐	☐	☐	☐	☐
Online based instructions for use (IFU)	☐	☐	☐	☐	☐	☐
Online videos	☐	☐	☐	☐	☐	☐
Online help ‘chat’ support	☐	☐	☐	☐	☐	☐
Telephone or video conference support	☐	☐	☐	☐	☐	☐

24. How much technical support do you think your patients would need in order to use these devices and data?

- None, they could work it out
- A one off training session
- Optional daily support
- Optional weekly support
- Ad hoc support

Page 8: Closing remarks

25. Do you have any thoughts or comments about the use of RMT, mHealth or patient worn devices that you think are important/useful to this study? If so please write them below.

26. Please select which charity you would like to receive the donation for your participation. Please select one.

- Multiple Sclerosis Society
- Epilepsy Action
- Mind (Mental Health Charity)

Page 9: Thank you

Thank you very much for your valuable contribution to this research project.

Your time spent on this will help us work towards understanding how these novel technologies might fit into clinical practice and what value they will provide to patients and healthcare professionals.

Please visit https://radar-cns.org to keep up to date with the study!

Kind Regards

RADAR-CNS Team
